# Infusions of Herbal Blends as Promising Sources of Phenolic Compounds and Bioactive Properties

**DOI:** 10.3390/molecules25092151

**Published:** 2020-05-04

**Authors:** Tiane C. Finimundy, Carla Pereira, Maria Inês Dias, Cristina Caleja, Ricardo C. Calhelha, Marina Sokovic, Dejan Stojković, Ana Maria Carvalho, Eduardo Rosa, Lillian Barros, Isabel C. F. R. Ferreira

**Affiliations:** 1 Centro de Investigação de Montanha (CIMO), Instituto Politécnico de Bragança, Campus de Santa Apolónia, 5300-253 Bragança, Portugal; tiane@ipb.pt (T.C.F.); carlap@ipb.pt (C.P.); maria.ines@ipb.pt (M.I.D.); ccaleja@ipb.pt (C.C.); calhelha@ipb.pt (R.C.C.); anacarv@ipb.pt (A.M.C.); 2 CITAB—University of Trás-os-Montes and Alto Douro (UTAD), Department of Agronomy, 5001-801 Vila Real, Portugal; erosa@utad.pt; 3 Institute for Biological Research “Siniša Stanković”, National Institute of Republic of Serbia, University of Belgrade, 11000 Belgrade, Serbia; mris@ibiss.bg.ac.rs (M.S.); dejanbio@ibiss.bg.ac.rs (D.S.)

**Keywords:** herbal blends, nutritional profile, phenolic compounds, HPLC-DAD/ESI-MSn, bioactive properties

## Abstract

Several plants have been used for medicinal applications and have been traditionally consumed as decoctions and infusions. Although some herbs are used alone as a beverage, they are often blended in mixtures to maximize their effects. Herein, the nutritional characterization of six infusions from herbal blends was evaluated using the official methods of analysis (AOAC international). A further characterization of the individual phenolic profile was also performed by HPLC-DAD/ESI-MSn, and finally bioactive potential was determined by evaluating the antioxidant, cytotoxic, anti-inflammatory, and antimicrobial activities of each blend. The wide variety of plants in each sample led to variability in the results for all analyzed parameters. However, blends containing 15% *Laurus nobilis* L. and 15% *Juglan regia* L. in their composition showed higher sugar content and energy contribution; higher concentration of phenolic compounds (phenolic acids and flavonoids); greater antioxidant, cytotoxic, and anti-inflammatory capacity; and also better antimicrobial effects against all the tested bacterial and fungal strains. Further studies will be necessary to evaluate the real synergistic effects that these two species show in the presence of other plants, and to evaluate their potential for application in various food, pharmaceutical, and nutraceutical products as infusion preparations.

## 1. Introduction

The search for new, safer, and sustainable high-added-value compounds relies to a certain degree on the continuous study of traditionally used medicinal plants [1]. These plants have been used since ancient times for their countless benefits, being associated with health-promoting properties [2,3]. Conventionally prepared as an infusion, decoction, or by maceration (also used for culinary purposes due to their fragrance and flavor), these herbs can be blended in different combinations of leaves, roots, barks, stems, and flowers, among other plant materials [4]. One of the botanical families containing an enormous range of species most commonly consumed as herbal tea is the Lamiaceae family, which brings together an enormous range of plants (e.g., *Rosmarinus officinalis* L. and *Thymus mastichina* L.) that have several recognized bioactive properties and beneficial health effects [5,6,7]. Another widely used botanical family is the Asteraceae family, comprising plants such as *Calendula arvensis* L. and *Chamaemelum nobile* L., which have been extensively studied [8,9,10] and also present a wide range of bioactive properties. The species I this family are of great commercial and economic value, with potential to be applied in the food and pharmaceutical industries. There are other plant families that are still underexplored, despite showing promising potential to be used as sources of high-added-value compounds, such as Lauraceae (e.g., *Laurus nobilis* L.), Oleaceae (e.g., *Olea europaea* L.), Vitaceae (e.g., *Vitis vinifera* L.), Apiaceae (e.g., *Foeniculum vulgare* Mill.), and Juglandaceae (e.g., *Juglans regia* L.) families, among others [11,12,13,14,15]. The demand of today’s consumers for foods marketed as healthier and natural has led the food industry to seek new formulation products to meet their high expectations [16], such as blending herbs from different botanical families. These mixtures can present additive and synergistic effects and increase the content of compounds of interest, improving the nutritional value of these products, and also by acting as sources of functional ingredients to be incorporated into food products [16,17]. In this sense, the present work was performed to chemically characterize six herbal blends (complete description in Table 1), namely relating to their profiles in individual sugars and phenolic compounds. Moreover, it also aimed to evaluate their antioxidant, cytotoxic, anti-inflammatory, and antimicrobial properties, comparing these results with previously reported data on the individual plants, allowing a better understanding of the eventual synergistic effects existing in these innovative mixtures.

## 2. Results and Discussion

### 2.1. Nutritional Analysis and Sugar Composition

The total contents of fat, ash, and proteins in the infused blend were, as expected (and confirmed in previous studies by Caleja et al. [18]), below the limits of detection, and consequently below the limits of quantification of the equipment and protocols used to perform these analyses. Therefore, assuming total sugars as total carbohydrates, the calculation of energy was performed according to the following equation: energy (cal) = 4 × (mg carbohydrates). Data on the free sugar content and energy contribution of the six infusions are shown in Table 2.

The qualitative and quantitative profiles of individual sugars in the six blends were quite different, with mix 1, mix 2, and mix 4 presenting fructose and glucose, and mix 3 and mix 6 only revealing the presence of fructose and sucrose, respectively. On the other hand, mix 5 was the only blend presenting these three sugars, also showing the highest concentration of total sugars (75 ± 2 mg/100 mL). Given the fact that this was the only blend containing *O. vulgare* and that it was its main constituent (60%), its high sugar content can be possibly ascribed to this species. In a previous study performed by Pereira et al. [19], a sample of *O. vulgare*, in which the subspecies was not identified, revealed a total sugar content of 19 ± 1 g/100 g of dry plant, also revealing the presence of trehalose; however, in refenced study the infusion was not assessed, so the results are not comparable to the ones presented herein. In mix 1, the most abundant species was *R. officinalis* (50%). For this herbal infusion, no sugars were detected in a previous study [19], which suggests that the sugar content of the mixture is due to the contributions of the other plants (*C. nobile*, *L. nobilis*, and *J. regia*). Indeed, Pereira et al. [19] reported a total sugar content of 15.0 ± 0.2 mg/100 mL for the *C. nobile* infusion and 10.46 ± 0.02 g/100 g of dried sample (not directly comparable) for *L. nobilis*. Regarding mix 2, in a previous study, *F. vulgare* infusion had a total sugar amount of 15.0 ± 0.9 mg/100 mL [19], lower than the one found for this mixture, which was composed of 40% of this plant. This suggests that the remaining plants in the blend had a higher contribution. Given the fact that *S. nigra* and *H. perforatum* were not present in the other herbal blends, this possibility was not discussed further.

Consequently, mix 5 also presented the highest energetic contribution (298 ± 6 cal/100 mL), followed by mix 1 (139 ± 4 cal/100 mL) and mix 2 (122 ± 5 cal/100 mL). As far as we know, there are no reports on these plants’ infusion energy values, except for *C. nobile*, *F. vulgare*, *M. pulegium*, *R. officinalis*, and *T. mastichina* individually (59.8 ± 0.9, 60 ± 3, 47.4 ± 0.85, ~0, and 34 ± 2 cal/100 mL, respectively) [19].

### 2.2. Phenolic Compounds Characterization

The retention time (Rt), wavelengths of maximum absorption in the visible region (λ_max_), mass spectral data, and tentative identification of the phenolic compounds in the six blends are presented in Table 3. The quantification results (mg/g extract) of the phenolic compounds present in the six blends are presented in Table 4. Fifty-two phenolic compounds were found in the six blends, among which twenty-eight were phenolic acids, twenty-three were flavonoids, and two were unknown compounds. All the plants in these blends were previously studied for their phenolic composition profiles. As such, the identification of all the compounds was performed using the bibliographic references described in the footnotes of Table 3. Twenty phenolic compounds were detected in mix 1, with peak 30 representing the main compound (luteolin-3’-*O*-glucuronide, 4.6 ± 0.1 mg/g extract). Additionally, this mixture had a high percentage of *R. officinalis*; its main phenolic compounds were not the ones described for the aqueous form of this plant (infusions and decoction) [20] or for hydroethanolic extracts [6], namely rosmarinic acid and its derivatives. In the case of the mixture, *C. nobile* [8] and *L. nobilis* [21] seemed to have a greater influence on the phenolic composition, with luteolin-*O*-glucuronide being the major peak detected. Tuberonic acid hexoside (peak 7, [M − H]^−^ at *m/z* 387) showed fragments at *m*/*z* 207, which corresponds to the aglycone after loss of hexose [M−H−162]^−^, and has been previously described in other plants of the Lamiaceae family [22]. Nine compounds were tentatively identified in mix 2. This blend comprised *F. vulgare*, *S. nigra*, and *H. perforatum*, and previous studies on the individual phenolic profiles of these plants revealed quercetin glycosylated derivatives as the main compounds [14,15,23], which is in accordance with the results obtained herein, showing peak 20 (quercetin-3-*O*-rutinoside, [M − H]^−^ at *m/z* 609) as the main compound (31.1 ± 1.3 mg/g extract). In mix 3, eight compounds were tentatively identified, with oleuropein (peak 44, [M − H]^−^ at *m/z* 539) being the major compound found. This is probably due to the 25% content of *M. pulegium*, since these secoiridoid-type compounds are very common and abundant in the *Oleaceae* family [12,24]. Thirteen phenolic compounds were tentatively identified in mix 4, with rosmarinic acid being the major compound (13.03 ± 0.05 mg/g extract). Taking into account the high amount of *M. cervina* in the mixture, and considering a study describing rosmarinic acid as the major compound found in this plant [25], it is possible to state that *M. cervina* has a great influence on the phenolic composition of this blend. Regarding mix 5, twelve phenolic compounds were tentatively identified, and as in the previous mixture, rosmarinic acid was the major compound (33.9 ± 0.1 mg/g of extract). Likewise, the high percentage (60%) of *O. vulgare* should explain this result, since *Origanum* genus plants are characterized by containing rosmarinic acid and its derivatives as the main compounds [26]. Finally, ten compounds were tentatively identified in mix 6, with peak 31 ([M − H]^−^ at *m/z* 447) being tentatively identified as kaempferol-*O*-hexoside, the major compound in this mix (35 ± 1 mg/g extract). This was an expected result, since the presence of this compound was described in the individual assessment of the phenolic composition of *T. mastichina* [6], *L. nobilis* [21], and *J. regia* [27]. 

Regarding the total compositions of phenolic compounds, it was possible to verify that mix 6, mix 5, and mix 2 presented higher contents of total phenolic compounds (72 ± 1, 69.6 ± 0.2, and 65 ± 2 mg/g extract, respectively). In mix 5, the total phenolic compound quantity is mainly due to the presence of phenolic acids (67.1 ± 0.2 mg/g extract), which represents 96% of the total phenolic composition. In mix 6, the total phenolic compound quantity is mainly due to the total flavonoid content (47.1 ± 0.2 mg/g extract), which represents 65.4% of the total composition. 

### 2.3. Bioactive Properties

The results for antioxidant, anti-inflammatory, and cytotoxic activities are shown in Table 5.

For TBARS assays, mix 1 and mix 6 presented the lowest IC_50_ values (4.5 ± 0.2 and 6.9 ± 0.3 µg/mL, respectively), which indicates high antioxidant activity. In fact, mix 1 revealed a higher lipid peroxidation inhibition capacity than the positive control, Trolox (5.8 ± 0.6 µg/mL). This activity could be explained by the presence of *R. officinalis*, reported in the literature as an excellent antioxidant plant, which is even applied in foodstuffs such as cottage cheese to increase shelf life [6]. Regarding the good results found for mix 6, the fact is that this blend presented the highest concentration of phenolic compounds, which are often reported as the main compounds responsible for the bioactive properties of plants. In a previous study, *O. vulgare* infusion presented an IC_50_ value of 22.8 ± 0.5 µg/mL, which was a high concentration when compared to 9 ± 1 µg/mL, the IC_50_ value obtained for mix 5 (with 60% of this plant), thus suggesting synergistic effects among the blended plants [26].

In terms of antihemolytic activity, the blend revealing the best results was mix 5, with an IC_50_ value of 4.0 ± 0.6 µg/mL. In fact, this blend presented the highest concentration of phenolic acids, which could be related to its antioxidant properties. All blends, with the exception of mix 2, revealed lower IC_50_ values than the positive control (85 ± 2 µg/mL). Studies developed by Caleja et al. [42] and Ribeiro et al. [43] proved the high antioxidant and antimicrobial potential of *F. vulgare* (present in mix 2) and *R. officinalis* (present in mix 1) and their capacity to increase the shelf life of functionalized foods. Moreover, in a previous study, *S. nigra* (present in mix 2) also revealed antioxidant properties (DPPH and FRAP assays) [44]. It is also reported in the literature that the combination of several plants in a drink beverage can provide a synergistic effect in terms of bioactivities, as their combination improves the antioxidant status and reduces oxidative stress [45].

Regarding cytotoxic activity, except for mix2 and mix 4, all blends revealed the capacity to inhibit the growth of the studied tumor cell lines in concentrations ranging from 175 ± 18 (mix 6 in HepG2) to 320 ± 12 (mix 3 in NCI-H460) µg/mL. Another positive aspects is that none of the mixtures revealed toxicity for non-tumor cells up to 400 µg/mL.

Similar observations could be made for anti-inflammatory activity, with mix 2 and mix 4 being the only blends not showing activity. Among the remaining infusions, mix 6 revealed the highest activity at a concentration of 262 ± 17 µg/mL. Considering the results reported by Dias et al. [11] and Vieira et al. [27], where *L. nobilis* and *J. regia* presented cytotoxic activity, in the present study it was expected that mix 2 and mix 4, containing these plants, would also present these properties, which was not observed. This was possibly due to the fact that such blends also included other species.

Finally, Table 6 shows the antimicrobial capacity of the six infused blends. It is possible to confirm the excellent antimicrobial potential of all mixtures against the analyzed microorganisms. *S. aureus* and *P. funiculosum* seemed to be the most sensitive microorganisms tested, with the infusions revealing lower minimal inhibitory concentration (MIC) and minimal bactericidal concentration/minimal fungal concentration (MBC/MFC) values than the positive controls. Among the tested blends, mix 1 revealed the highest antibacterial properties, with inhibitory and bactericidal concentrations ranging between 0.25 and 2 mg/mL. These results are in accordance with the ones obtained for *R. officinalis* (50% of the composition of mix 1) hydroethanolic extracts, which showed promising antimicrobial capacity [41]. On the other hand, mix 6 revealed the greatest antifungal activity, being able to inhibit fungal growth at 0.12 to 0.5 mg/mL and presenting fungicidal capacity in concentrations ranging from 0.25 to 5 mg/mL. This blend was more effective than the positive controls for all the tested fungi, except in terms of killing *A. fumigatus*. These results are in accordance with the ones obtained for *Thymus* sp. (70% of the composition of mix 6), which has been prescribed for the treatment of infectious diseases [33]. The antimicrobial capacity of *Thymus* sp. infusion has often been correlated with the presence of flavonoids and phenolic acids [6,21,46].

## 3. Materials and Methods

### 3.1. Samples and Infusions Preparation

Each dry plant material used to prepare the blends was provided by Ervital^®^ (company based in Castro Daire, Portugal). The botanical identification of the fifteen samples was confirmed by Professor Doctor Ana Maria Carvalho (Polytechnic Institute of Bragança, Trás-os-Montes, Portugal) and consisted of: (i) flowering aerial parts of *Foeniculum vulgare* Mill., *Hypericum perforatum* L., *Mentha cervina* L., *Mentha pulegium* L., *Origanum vulgare* subs. *virens* Hoffm. and Link, and *Thymus mastichina* L; (ii) flower heads of *Calendula arvensis* L., *Chamaemelum nobile* (L.) All., and *Sambucus nigra* L.; and (iii) leaves of *Juglans regia* L., *Laurus nobilis* L., *Olea europaea* L., *Rosmarinus officinalis* L., *Rubus idaeus* L., and *Vitis vinifera* L. The six herbal mixtures (mix 1 to mix 6) were blended based on their folk uses and sensory characteristics, following the proportions and traditional combinations of such species: Mix 1: 50% *R. officinalis*, 20% *C. nobile*, 15% *L. nobilis*, and 15% *J. regia*.; mix 2: 40% *F. vulgare*, 30% *S. nigra*, and 30% *H. perforatum*; mix 3: 50% *M. pulegium*, 25% *O. europaea*, and 25% *V. vinifera*; mix 4: 60% *M. cervine*, 20% *C. arvensis*, and 20% *R. idaeus*; mix 5: 60% *O. vulgare*, 10% *C. nobile*, 15% *L. nobilis*, and 15% *J. regia*.; mix 6: 70% *T. mastichina*, 15% *L. nobilis*, and 15% *J. regia*. All the samples were reduced to a fine powder and protected from light and humidity until further analysis. Furthermore, the preparation of infusions was based on the protocol previously described by Pereira et al. [47] and following particular extraction conditions for each blend: mix 1: 10 g/L, 95 °C, 5–7 min; mix 2: 10 g/L, 90 °C, 4–6 min; mix 3: 4 g/L, 95 °C, 5–8 min; mix 4: 4 g/L, 90 °C, 4–6 min; mix 5: 10 g/L, 90 °C, 5–7 min; and mix 6: 10 g/L, 90 °C, 5–7 min. All samples were filtered through Whatman # 4 paper, frozen at −20 °C, and then lyophilized. The extracts were protected from light and humidity until further analysis.

### 3.2. Nutritional Analysis and Sugar Content

Fat, carbohydrate, ash, and protein contents of the six dry infusion extracts were analyzed following the AOAC [48] procedures. Free sugars were analyzed following the method previously described by Barros et al. [28], using HPLC coupled to a refractive index detector (Knauer, Smartline 1000 and Smartline 2300 systems, respectively) and melezitose as internal standard. The results were expressed in mg/100 mL of infusion. Finally, the energetic value was calculated according the equation: energy (cal) = 4 × (mg proteins + mg carbohydrates) + 9 × (mg lipids).

### 3.3. Phenolic Compounds Composition

The dry infusion extracts were resuspended in water at a concentration of 10 mg/mL. The phenolic profile was determined by liquid chromatography (Dionex Ultimate 3000 UPLC, Thermo Scientific, San Jose, CA, USA) with a diode array detector (280, 330, and 370 nm wavelengths) equipped with an ESI source and working in negative mode (Linear Ion Trap LTQ XL, Thermo Scientific, San Jose, CA, USA) [49]. Chromatographic separation was achieved with a Waters Spherisorb S3 ODS-2C18 (3 m, 4.6 mm × 150 mm, Waters, Mil-ford, MA, USA) column thermostat at 35 °C. The solvents used were: (A) 0.1% formic acid in water, (B) acetonitrile. The established isocratic elution gradient was 15% B (5 min), 15% B to 20% B (5 min), 20–25% B (10 min), 25–35% B (10 min), 35–50% B (10 min), and re-equilibration of the column, using a flow rate of 0.5 mL/min [49]. The phenolic compounds were identified by comparing their retention times, UV, and mass spectra values with those obtained from standard compounds and with the literature. For quantitative analysis, 7-level calibration curves prepared with appropriate standards were used. The results were expressed in mg per g of dry extract (mg/g) as mean ± standard deviation of three independent analyses.

### 3.4. Evaluation of Bioactive Properties

#### 3.4.1. Antioxidant Activity

All samples were tested by two in vitro assays. The thiobarbituric acid reactive species (TBARS) assay uses a brain porcine homogenate measured by spectrophotometry at 532 nm [28]. This assay is used to determine the TBARS content in various samples, including those used to detect a sort of protective activity (usually antilipoperoxidant activity) using a fat or membrane model and an oxidant to induce the damage. The oxidative hemolysis inhibition (OxHLIA) assay is used to evaluate the antihemolytic activity of the extracts using sheep erythrocytes, measured by spectrophotometry at 690 nm [50]. Hemolysis was previously induced using 2,2’-Azobis(2-amidinopropane) dihydrochloride (AAPH). The results were expressed as EC_50_ values (sample concentration providing 50% of antioxidant activity, shown in µg/mL), and Trolox was used as a positive control for both assays.

#### 3.4.2. Anti-inflammatory Activity

The anti-inflammatory activity was assessed following a procedure described by Svobodova et al. [51]. The dried extracts were re-dissolved in water at 8 mg/mL and evaluated in mouse lipopolysaccharide (LPS)-stimulated macrophage-like cell line RAW 264.7. The results were expressed as IC_50_ values (sample concentration providing 50% of anti-inflammatory activity, μg/mL) and dexamethasone (50 μM) was used as a positive control.

#### 3.4.3. Cytotoxic Activity in Tumor and Non-Tumor Cells

The cytotoxic potential was evaluated in four different human tumor cell lines (HeLa (cervical carcinoma), HepG2 (hepatocellular carcinoma), MCF-7 (breast adenocarcinoma), and NCI-H460 (non-small-cell lung cancer)) and a primary culture of non-tumor cells (PLP2 (porcine liver)). To monitor the growth of cell cultures, which were sub-cultured and plated in 96-well plates (density of 1.0 × 10^4^ cells/well), a phase-contrast microscope was used, following the protocol defined by Guimarães et al. [8]. Ellipticin was used as a positive control, and the results were expressed as GI_50_ values (sample concentration that inhibited 50% of cell growth, μg/mL).

#### 3.4.4. Antimicrobial Activity

The antibacterial activity was evaluated using Gram (+) bacteria (*Bacillus cereus* (food isolate), *Listeria monocytogenes* (NCTC 7973), *Staphylococcus aureus* (ATCC 6538), and *Micrococcus flavus* (ATCC 10240)), as well as Gram (−) bacteria (*Enterobacter cloacae* (human isolate) and *Salmonella* Typhymurium (ATCC 13311)), following a protocol previously described by Soković et al. [46]. On the other hand, *Aspergillus niger* (ATCC 6275), *Aspergillus versicolor* (ATCC 11730), *Aspergillus fumigatus* (ATCC 9197), *Penicillium funiculosum* (ATCC 36839), and *Penicillium aurantiogriseum* (food isolate) were used to evaluate the antifungal activity, following the protocol described by Soković and Van Griensven [52]. Sodium sulphite (E221) and potassium metabisulphite (E224) food additives were used as positive controls for both activities. The microorganisms are deposited at the Institute for Biological Research “Siniša Stanković”, National Institute of Republic of Serbia, University of Belgrade. The results were expressed as minimal inhibitory concentration (MIC), minimal bactericidal concentration (MBC), and minimal fungicidal concentration (MFC) values.

### 3.5. Statistical Analysis

For each herbal blend, three individual samples were analyzed, and all assays were prepared in triplicate. The results were analyzed using one-way analysis of variance (ANOVA), followed by Tukey’s HSD test as *p* = 0.05, and are expressed as mean values with standard deviation (SD). When less than three results were present in each individual analysis, Student’s *t*-test *p*-values were used to determine the significant difference, with *p* = 0.05. Both of these statistical treatments were carried out using the SPSS v.22.0 program.

## 4. Conclusions

The infusions of different blended species proved to be valuable sources of high-added-value compounds and also showed high capacity for bioactive activity. Given the wide variety of species in each sample, a great variability in results was also expected, rather than obtaining a mixture that stood out in terms of all the analyzed parameters. However, mix 5 had higher contents of soluble sugar, energy contribution, total phenolic acids, and a higher capacity to inhibit erythrocyte hemolysis and the proliferation of the HeLa tumor cell line. Mix 6 presented the highest content of total phenolic compounds (especially flavonoids), higher capacity to inhibit the growth of HepG2 tumor cell line, higher anti-inflammatory activity, and higher antifungal activity. Finally, mix 1 revealed a higher capacity to inhibit lipid peroxidation in the TBARS assay, higher capacity to inhibit the growth of the MCF-7 tumor cell line, and higher antibacterial potential, presenting the lowest MIC and MBC values for Gram-positive and Gram-negative bacteria (even lower than the positive controls). The fact that blends 1, 5, and 6 contain 15% *L. nobilis* and 15% *J. regia* in their composition could explain the better results obtained with these plant mixtures compared to the other ones that did not contain any of these species. As stated earlier, *L. nobilis* and *J. regia* have already been described as highly promising plants for obtaining high-added-value compounds with bioactive potential. Future studies will be necessary to evaluate the real synergistic effects presented by these two plants in the presence of others, and to evaluate their potential for further application in various food, pharmaceutical, and nutraceutical products.

## Figures and Tables

**Table 1:** Description and morphological characteristics of the six herbal blends provided by Ervital^®^ company.

**Mix 1**	**Mix 2**	**Mix 3**
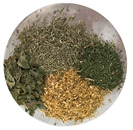	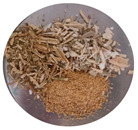	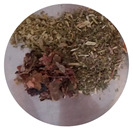
50% *R. officinalis*, 20% *C. nobile*, 15% *L. nobilis, 1*5% *J. regia*	40% *F. vulgare*, 30% *S. nigra*, 30% *H. perforatum*	50% *M. pulegium*, 25% *O. Europea*, 25% *V. vinifera*
**Mix 4**	**Mix 5**	**Mix 6**
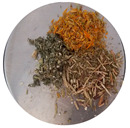	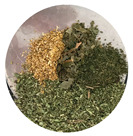	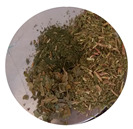
60% *M. cervine*, 20% *C. arvensis* 20%, *R. idaeus*	60% *O. vulgare*, 10% *C. nobile*, 15% *L. nobilis*, 15% *J. regia*	70% *T. mastichina*, 15% *L. nobilis*, 15% *J. regia*

The morphological characteristics of the six herbal blends were flowering aerial parts of *Foeniculum vulgare* Mill., *Hypericum perforatum* L., *Mentha cervina* L., *Mentha pulegium* L., *Origanum vulgare* subs. *virens* Hoffm. and Link, and *Thymus mastichina* L; flower heads of *Calendula arvensis* L., *Chamaemelum nobile* (L.) All., and *Sambucus nigra* L.; and leaves of *Juglans regia* L., *Laurus nobilis* L., *Olea europaea* L., *Rosmarinus officinalis* L., *Rubus idaeus* L., and *Vitis vinifera* L.

**Table 2:** Free sugar and carbohydrate contents and energy contributions of the six infused blends (mean ± SD).

	Mix 1	Mix 2	Mix 3	Mix 4	Mix 5	Mix 6
Free sugars (mg/100 mL)						
Fructose	22.1 ± 0.4 ^b^	13.3 ± 0.6 ^c^	9.1 ± 0.4 ^d^	13.3 ± 0.3 ^c^	30 ± 1 ^a^	nd
Glucose	12.6 ± 0.5 ^c^	17.1 ± 0.7 ^a^	nd	3.5 ± 0.1 ^d^	15.3 ± 0.5 ^b^	nd
Sucrose *	nd	nd	nd	nd	29 ± 1	6.5 ± 0.1
Total sugars	35 ± 1 ^b^	30 ± 1 ^c^	9.1 ± 0.4 ^e^	16.8 ± 0.1 ^d^	75 ± 2 ^a^	6.5 ± 0.1 ^f^
Energy (cal/100 mL)	139 ± 4 ^b^	122 ± 5 ^c^	36 ± 1 ^e^	67.2 ± 0.6 ^d^	298 ± 6 ^a^	25.8 ± 0.3 ^f^

Protein, ash, and fat contents were zero; results expressed as medium value ± standard deviation (SD); nd = not detected. Mix 1: 50% *R. officinalis*, 20% *C. nobile*, 15% *L. nobilis*, 15% *J. regia*. Mix 2: 40% *F. vulgare*, 30% *S. nigra*, 30% *H. perforatum*. Mix 3: 50% *M. pulegium*, 25% *O. europaea*, 25% *V. vinifera*. Mix 4: 60% *M. cervine*, 20% *C. arvensis*, 20% *R. idaeus*. Mix 5: 60% *O. vulgare*, 10% *C. nobile*, 15% *L. nobilis*, 15% *J. regia*. Mix 6: 70% *T. mastichina*, 15% *L. nobilis*, 15% *J. regia.* The statistical treatment was performed by comparing the mixtures; therefore, in each row different letters indicate statistically significant differences (*p* < 0.05). * For sucrose individually, the statistical analysis was performed using the Student’s *t*-test *p*-value, <0.001.

**Table 3:** Retention time (Rt), wavelengths of maximum absorption in the visible region (λ_max_), mass spectral data, and identification of phenolic compounds in the six infused blends.

Peak	Rt (min)	λmax (nm)	[M − H] *m*/*z*	MS^2^	Tentative Identification
**1**	4.47	328	311	179(100),149(83),135(72)	Caftaric acid
**2**	4.58	324	353	191(100),179(45),135(7)	3-*O*-Caffeoylquinic acid
**3**	5.34	319	305	226(15),175(6),135(40)	Epigallocatechin
**4**	6.45	319	353	191(20),179(50),173(100),135(3)	4-*O*-Caffeoylquinic acid
**5**	6.48	267	305	226(13), 225(100),175(2), 97(44)	(+)-Gallocatechin
**6**	6.81	319	353	191(100),179(24),173(37)	5-*O*-Caffeoylquinic acid
**7**	7.88	322	387	207(100),163(20)	Tuberonic acid glucoside
**8**	8.8	278	617	287(100)	Eriodictyol-7-*O*-rutinoside
**9**	9.59	320	179	135(100)	Caffeic acid
**10**	9.8	319	313	197(100)	Salvianolic acid F
**11**	10.74	284	449	287(100)	Eriodictyol-*O*-hexoside isomer 1
**12**	11.71	340	337	191(3),173(100),163(43),155(12),119(12)	4-*p*-Coumaroylquinic acid
**13**	12.2	284	449	287(100)	Eriodictyol-*O*-hexoside isomer 2
**14**	13.33	339	637	285(100)	Luteolin-*O*-di-glucuronide
**15**	14.25	333	473	311(19),293(19),149(100),135(28)	Chicoric acid
**16**	14.28	343	537	493(7),339(100),295(90)	Salvianolic acid A isomer 1
**17**	14.4	310	537	493(100),359(33),313(5),295(3)	Lithospermic acid A
**18**	14.72	327	555	537(3),511(3),493(39),311(10),269(20),197(36),179	Salvianolic acid K
**19**	15.1	333	521	359(50),197(20),179(37),161(100)	Rosmarinic acid hexoside
**20**	15.31	334	609	301(100)	Quercetin-3-*O*-rutinoside
**21**	15.58	344	463	301(100)	Quercetin-*O*-hexoside
**22**	15.62	335	491	311(100),293(20),197(12)	Salvianolic acid C
**23**	15.96	332	797	779(100),599(42),555(50),359(37),313(12), 169(5)	Unknown
**24**	16.66	351	769	315(100), 300(10)	Isorhamentin-3-*O*-rhamnosyl-rutinoside
**25**	17.02	332	421	153(100)	4-[[(2′,5′Dihydroxybenzoyl)oxy]methyl]phenyl-*O*-β-d-glucopyranoside
**26**	17.21	272, 324sh	539	495(13), 359(21), 297(100), 279(64), 197(34), 179(36), 161(34), 135(18)	Yunnaneic acid D isomer
**27**	17.41	308	609	301(100)	Quercetin-*O*-rutinoside
**28**	17.9	347	477	301(100)	Quercetin-*O*-glucuronide
**29**	18.18	344	717	519(100),493(8),339(39),321(92),295(23),197(3)	Salvianolic acid B isomer 1
**30**	18.45	346	461	285(100)	Luteolin-3’-*O*-glucuronide
**31**	18.81	343	447	285(100)	Kaempferol-*O*- hexoside
**32**	19.86	337	477	315(100), 300(19)	Isorhamnetin-3-*O*-glucoside
**33**	19.9	370	549	505(100),301(74)	Quercetin-7-*O*-malonylhexoside
**34**	19.97	331	783	513(100), 497(10), 351(3), 289(55), 245(4)	Catechin derivative
**35**	20.24	334	717	519(100),493(8),339(39),321(92),295(23),197(3)	Salvianolic acid B isomer 2
**36**	20.64	343	593	285(100)	Kaempferol-3-*O*-rutinoside
**37**	21.56	330	359	197(98), 179(94), 161(100), 135(58)	*cis*-Rosmarinic acid
**38**	21.65	344	623	315(100),300(10)	Isorhamnetin-3-*O*-rutinoside
**39**	22.43	330	719	539(12), 521(10), 359(65), 197(6), 179(8), 161(17), 135(3)	Sagerinic acid
**40**	22.54	328	717	519(100), 339(27), 321(87), 295(13), 277(33)	Salvianolic acid L
**41**	22.68	325	555	493(50),292(100),197(36),179(29)	Salvianolic acid K
**42**	22.68	330	359	197(98), 179(94), 161(100), 135(58)	*trans*-Rosmarinic acid
**43**	23.06	337	477	315(100), 300(19)	Isorhamnetin-3-*O*-glucoside
**44**	23.69	340	539	377(100), 307(41),275(31)	Oleuropein
**45**	23.75	329	549	505(10), 463(25), 301(100)	Quercetin-*O*-malonylhexoside
**46**	24.63	346	461	285(100)	Luteolin-3’-*O*-glucuronide
**47**	25.45	331	537	493(100),359(33),313(5),295(3)	Lithospermic acid A
**48**	25.66	344	519	315(100)	Isorhamnetin-3-*O*-(6’-acetyl)-glucoside
**49**	28.43	327	503	285(100)	Acetylluteolin-*O*-glucuronide
**50**	28.89	331	503	285(100)	Acetylluteolin-*O*-glucuronide
**51**	30.39	323	493	359(84), 313(13), 295(58), 269(7), 197(31), 179(41),161(91),135(86)	Salvianolic acid A isomer 2
**52**	30.74	331	503	285 (100)	Acetylluteolin-*O*-glucuronide

References used for identification: [6] for peaks 4 and 47; [9] for peaks 2, 28, 33, 45, and 46; [28] for peak 10; [21] for peaks 5 and 38; [22] for peak 7; [29] for peaks 1, 9, 15, 17, 18, 20, 21, 27, 36, and 41; [30] for peak 3; [31] for peaks 6, 25, and 31; [32] for peak 8; [33] for peaks 11, 13, and 30; [27] for peak 12; [34] for peak 14; [35] for peak 16; [25] for peaks 19, 24, 26, 32, 37, 39, 42, and 48; [36] for peak 22; [37] for peaks 29 and 35; [38] for peak 34; [39] for peaks 40 and 51; [40] for peak 43; [24] for 44; [41] for peaks 49, 50, and 52.

**Table 4:** Quantification of the phenolic compounds present in the six infused blends (mean ± SD; mg/g extract).

Peak	Compounds	Mix 1	Mix 2	Mix 3	Mix 4	Mix 5	Mix 6
**1**	Caftaric acid	1.60 ± 0.01	nd	1.8 ± 0.2	nd	nd	nd
**2**	3-*O*-Caffeoylquinic acid	nd	3.2 ± 0.2 ^a^	nd	2.15 ± 0.02 ^d^	2.56 ± 0.05 ^c^	2.7 ± 0.1 ^b^
**3**	Epigallocatechin	0.066 ± 0.003	nd	nd	1.3 ± 0.1	nd	nd
**4**	4-*O*-Caffeoylquinic acid	0.9 ± 0.1 ^c^	1.6 ± 0.1 ^a^	nd	nd	nd	1.2 ± 0.3 ^b^
**5**	(+)-Gallocatechin	2.5 ± 0.2	nd	nd	nd	nd	nd
**6**	5-*O*-Caffeoylquinic acid	nd	22.4 ± 0.2 ^a^	nd	nd	1.0 ± 0.1 ^c^	1.6 ± 0.2 ^b^
**7**	Tuberonic acid glucoside	0.216 ± 0.003	nd	nd	nd	nd	nd
**8**	Eriodictyol-7-*O*-rutinoside	nd	nd	nd	nd	0.69 ± 0.01	nd
**9**	Caffeic acid	0.231 ± 0.002	nd	nd	nd	nd	nd
**10**	Salvianolic acid F	nd	nd	nd	1.50 ± 0.01	nd	nd
**11**	Eriodictyol-*O*-hexoside isomer 1	nd	nd	nd	nd	nd	0.6 ± 0.1
**12**	4-*p*-Coumaroylquinic acid	0.33 ± 0.04	nd	nd	nd	nd	nd
**13**	Eriodictyol-*O*-hexoside isomer 2	nd	nd	nd	nd	nd	3.5 ± 0.6
**14**	Luteolin-*O*-di-glucuronide	nd	nd	nd	nd	1.891 ± 0.001	nd
**15**	Chicoric acid	nd	nd	3.5 ± 0.4	nd	nd	nd
**16**	Salvianolic acid A isomer 1	nd	nd	nd	nd	0.48 ± 0.01	nd
**17**	Lithospermic acid A	nd	nd	nd	1.12 ± 0.03	nd	nd
**18**	Salvianolic acid K	0.99 ± 0.01	nd	nd	nd	nd	nd
**19**	Rosmarinic acid hexoside	0.86 ± 0.03	nd	nd	nd	nd	nd
**20**	Quercetin-3-*O*-rutinoside	nd	31 ± 1	nd	0.20 ± 0.03	nd	nd
**21**	Quercetin-*O*-hexoside	nd	nd	nd	nd	nd	8.9 ± 0.1
**22**	Salvianolic acid C	0.82 ± 0.03	nd	nd	nd	nd	nd
**23**	Unknown	nd	nd	nd	nd	nq	nd
**24**	Isorhamentin-3-*O*-rhamnosyl-rutinoside	nd	nd	nd	2.51 ± 0.05	nd	nd
**25**	4-[[(2′,5′Dihydroxybenzoyl)oxy]methyl]phenyl-*O*-β-d-glucopyranoside	nd	nd	nd	nd	14.6 ± 0.2	nd
**26**	Yannaneic acid D isomer	nd	nd	nd	0.6 ± 0.1	nd	nd
**27**	Quercetin-*O*-rutinoside	nd	nd	0.62 ± 0.01	nd	nd	nd
**28**	Quercetin-*O*-glucuronide	nd	nd	1.98 ± 0.05	1.3 ± 0.1	nd	nd
**29**	Salvianolic acid B isomer 1	nd	nd	6.3 ± 0.5	nd	1.26 ± 0.05	nd
**30**	Luteolin-3’-*O*-glucuronide	4.6 ± 0.1	nd	nd	nd	nd	nd
**31**	Kaempferol-*O*- hexoside	nd	nd	nd	nd	nd	35 ± 1
**32**	Isorhamnetin-3-*O*-glucoside	1.166 ± 0.001	nd	nd	nd	nd	nd
**33**	Quercetin-7-*O*-malonylhexoside	nd	1.4 ± 0.2	nd	nd	nd	nd
**34**	Catechin derivative	nd	nd	nd	nd	nq	nd
**35**	Salvianolic acid B isomer 2	nd	nd	nd	0.95 ± 0.05	nd	nd
**36**	Kaempferol-3-*O*-rutinoside	nd	0.6 ± 0.2	nd	nd	nd	nd
**37**	*cis*-Rosmarinic acid	nd	nd	nd	13.03 ± 0.05 ^b^	33.9 ± 0.1 ^a^	11.826 ± 0.004 ^c^
**38**	Isorhamnetin-3-*O*-rutinoside	nd	2.7 ± 0.1	nd	nd	nd	nd
**39**	Sagerinic acid	2.29 ± 0.01 ^b^	nd	0.93 ± 0.01 ^c^	0.83 ± 0.05 ^d^	4.4 ± 0.1 ^a^	nd
**40**	Salvianolic acid L	nd	nd	0.9 ± 0.1	nd	nd	nd
**41**	Salvianolic acid K	nd	nd	nd	nd	nd	5 ± 1
**42**	*trans*-Rosmarinic acid	0.70 ± 0.01	nd	nd	0.9 ± 0.1	nd	nd
**43**	Isorhamnetin-3-*O*-glucoside	nd	1.08 ± 0.03	nd	nd	nd	nd
**44**	Oleuropein	nd	nd	9.0 ± 0.3	nd	nd	nd
**45**	Quercetin-*O*-malonylhexoside	0.558 ± 0.005	nd	nd	nd	nd	nd
**46**	Luteolin-3’-*O*-glucuronide	1.25 ± 0.03	nd	nd	nd	nd	nd
**47**	Lithospermic acid A	0.6 ± 0.1 ^c^	nd	nd	0.67 ± 0.03 ^c^	8.8 ± 0.1 ^a^	1.91 ± 0.01 ^b^
**48**	Isorhamnetin-3-*O*-(6’-acetyl)-glucoside	nd	0.90 ± 0.03	nd	nd	nd	nd
**49**	Acetylluteolin-*O*-glucuronide	0.57 ± 0.02	nd	nd	nd	nd	nd
**50**	Acetylluteolin-*O*-glucuronide	0.65 ± 0.04	nd	nd	nd	nd	nd
**51**	Salvianolic acid A isomer 2	nd	nd	nd	4.31 ± 0.05	nd	nd
**52**	Acetylluteolin-*O*-glucuronide	0.63 ± 0.04	nd	nd	nd	nd	nd
	**Total Phenolic Acids**	**9.50 ± 0.03 ^f^**	**27.2 ± 0.1 ^b^**	**22.4 ± 0.1 ^e^**	**26.1053 ± 0.0004 ^c^**	**67.1 ± 0.2 ^a^**	**25 ± 2 ^d^**
	**Total Flavonoids**	**12.1 ± 0.1 ^c^**	**38 ± 2 ^b^**	**3.5 ± 0.1 ^e^**	**5.29 ± 0.05 ^d^**	**2.58 ± 0.01 ^f^**	**47.1 ± 0.2 ^a^**
	**Total Phenolic Compounds**	**21.6 ± 0.1 ^f^**	**65 ± 2 ^c^**	**23.2 ± 0.2 ^e^**	**31.40 ± 0.05 ^d^**	**69.6 ± 0.2 ^b^**	**72 ± 1 ^a^**

Note: nd: not detected; nq: not quantified. Mix 1: 50% *R. officinalis*, 20% *C. nobile*, 15% *L. nobilis*, 15% *J. regia*. Mix 2: 40% *F. vulgare*, 30% *S. nigra*, 30% *H. perforatum*. Mix 3: 50% *M. pulegium*, 25% *O. europaea*, 25% *V. vinifera*. Mix 4: 60% *M. cervine*, 20% *C. arvensis*, 20% *R. idaeus*. Mix 5: 60% *O. vulgare*, 10% *C. nobile*, 15% *L. nobilis*, 15% *J. regia*. Mix 6: 70% *T. mastichina*, 15% *L. nobilis*, 15% *J. regia.* Calibration curves: chlorogenic acid (*y* = 168,823*x* – 161,172; *R*^2^ = 1.000; LOD = 0.20 µg/mL; LOQ = 0.68 µg/mL; peaks 1, 2, 4, 6, and 15); epicatechin (*y* = 10,314*x* + 147,331; *R*^2^ = 0.9998; LOD = 0.15 μg/mL; LOQ = 0.78 μg/mL; peaks 3 and 5); *p*-coumaric acid (*y* = 301,950*x* + 6966.7; *R*^2^ = 0.9999; LOD = 0.68 μg/mL; LOQ = 1.61 μg/mL; peaks 7 and 12); naringenin (*y* = 18,433x + 78,903; *R*^2^ = 0.9997; LOD = 0.17 µg/mL; LOQ = 0.81 µg/mL; peaks 8 and 13); caffeic acid (*y* = 388,345*x* + 406,369; *R*^2^ = 0.9991; LOD = 0.78 μg/mL; LOQ = 1.97 μg/mL; peaks 9 and 12); rosmarinic acid (*y* = 191,291*x* – 652,903; *R*^2^ = 0.999; LOD = 0.15 µg/mL; LOQ = 0.68 µg/mL; peaks 10, 16, 17, 18, 19, 22, 26, 29, 35, 37, 39, 40, 41, 42, 47, and 51); quercetin-3-*O*-glucoside (*y* = 34,843x – 160,173; *R*^2^ = 1.000; LOD 0.21 μg/mL; LOQ 0.71 μg/mL; peaks 14, 21, 28, 32, 33, 43, 45, 46, 48, 49, 50, and 52); quercetin-3-*O*-rutinoside (*y* = 13,343*x* + 76,751; *R*^2^ = 0.9998; LOD 0.18 μg/mL; LOQ 0.65 μg/mL; peaks 20, 24, 27, 30, 31, 36, and 38); protocatechuic acid (*y* = 214,168*x* + 27,102; *R*^2^ = 0.9997; LOD = 0.14 μg/mL; LOQ = 0.52 μg/mL; peak 25); oleuropein (*y* = 32,226*x* + 12,416; *R*^2^ = 0.9997; LOD = 0.69µg/mL and LOQ = 1.96 µg/mL; peak 44). The statistical treatment was performed by comparing the mixes; therefore, in each row different letters indicate statistically significant differences (*p* < 0.05). Mean statistical differences obtained by Student’s *t*-test for peaks 1, 3, 20, 28, 29, and 42 was <0.001.

**Table 5:** Antioxidant, anti-inflammatory, and cytotoxic activity of the six infused blends (mean ± SD).

	Mix 1	Mix 2	Mix 3	Mix 4	Mix 5	Mix 6
**Antioxidant activity (IC_50_, µg/mL)**						
TBARS	4.5 ± 0.2 ^f^	23.1 ± 0.3 ^a^	14 ± 1 ^c^	22 ± 1 ^b^	9 ± 1 ^d^	6.9 ± 0.3 ^e^
OxHLIA (Δt = 60 min)	22 ± 1 ^c^	106 ± 4 ^a^	9.7 ± 0.7 ^e^	12 ± 2 ^d^	4.0 ± 0.6 ^f^	35 ± 2 ^b^
**Cytotoxic activity (GI_50_, µg/mL)**						
MCF-7	209 ± 5 ^c^	>400	238 ± 4 ^b^	>400	236 ± 16 ^b^	254 ± 17 ^a^
NCI-H460	257 ± 10 ^b^	>400	320 ± 12 ^a^	>400	250 ± 13 ^b^	258 ± 16 ^b^
HeLa	246 ± 16 ^a^	>400	217 ± 11 ^c^	>400	213 ± 9 ^c^	227 ± 10 ^b^
HepG2	226 ± 13 ^b^	>400	304 ± 6 ^a^	>400	230 ± 25 ^b^	175 ± 18 ^c^
PLP2	>400	>400	>400	>400	>400	>400
**Anti-inflammatory activity (IC_50_, µg/mL)**						
RAW 246.7	321 ± 4 ^a^	>400	276 ± 12^b^	>400	276 ± 12 ^b^	262 ± 17 ^c^

Mix 1: 50% R. officinalis, 20% C. nobile, 15% L. nobilis, 15% J. regia. Mix 2: 40% F. vulgare, 30% S. nigra, 30% H. perforatum. Mix 3: 50% M. pulegium, 25% O. europaea, 25% V. vinifera. Mix 4: 60% M. cervine, 20% C. arvensis, 20% R. idaeus. Mix 5: 60% O. vulgare, 10% C. nobile, 15% L. nobilis, 15% J. regia. Mix 6: 70% T. mastichina, 15% L. nobilis, 15% J. regia. EC_50_ values corresponded to the extract concentration that inhibits 50% of the oxidation and inflammatory processes. Trolox (IC_50_ values): TBARS (thiobarbituric acid reactive species): 5.8 ± 0.6 µg/mL; OxHLIA (oxidative hemolysis inhibition, 60 min): 85 ± 2 µg/mL. Dexamethasone (IC_50_ values): 16 ± 1 µg/mL. GI_50_ values correspond to the concentration that causes 50% inhibition of cell proliferation. Note: MCF-7 = human breast adenocarcinoma; NCI-H460 = human lung carcinoma; HeLa = human cervix adenocarcinoma; HepG2 = hepatocellular carcinoma; PLP2 = primary culture of non-tumoral pig liver cells. Ellipticine (GI_50_ values): MCF-7: 1.21 ± 0.02 µg/mL; NCI-H460: 0.91 ± 0.11 µg/mL; HeLa: 1.03 ± 0.09 µg/mL; HepG2: 1.1 ± 0.09 µg/mL; PLP2: 2.29 ± 0.18 µg/mL. Raw 246.7 (Mouse lipopolysaccharide (LPS)-stimulated macrophage-like cell line). Results expressed as mean values ± standard deviation (SD). The statistical treatment was performed by comparing the mixtures; therefore, in each row different letters indicate statistically significant differences (*p* < 0.05).

**Table 6:** Antimicrobial activity of the six infused blends and positive controls.

	Mix 1		Mix 2		Mix 3		Mix 4		Mix 5		Mix 6		E221		E224	
**Antibacterial activity (mg/mL)**																
**Bacteria**	MIC	MBC	MIC	MBC	MIC	MBC	MIC	MBC	MIC	MBC	MIC	MBC	MIC	MBC	MIC	MBC
***Gram-negative***																
*Salmonella* Typhymurium	0.5	1	4	8	1	2	2	2	1	2	1	1	1	2	1	1
*Enterobacter cloacae*	0.5	1	2	2	1	2	1	2	1	2	1	2	2	4	0.5	0.5
***Gram-positive***																
*Bacillus cereus*	1	1	1	2	1	2	1	2	1	2	1	1	0.5	0.5	2	4
*Listeria monocytogenes*	0.5	1	2	2	1	2	1	2	1	2	1	2	1	2	0.5	1
*Staphylococcus aureus*	0.25	0.25	1	1	0.25	0.5	0.25	0.25	0.25	0.5	0.25	0.5	4	4	1	1
*Micrococcus flavus*	2	2	2	4	2	4	1	2	1	2	1	2	1	2	1	2
**Antifungal activity (mg/mL)**																
**Fungi**	MIC	MFC	MIC	MFC	MIC	MFC	MIC	MFC	MIC	MFC	MIC	MFC	MIC	MFC	MIC	MFC
*Aspergillus niger*	0.5	1	0.5	1	1	1	0.5	1	0.5	1	0.25	0.5	1	2	1	1
*Aspergillus versicolor*	0.5	1	0.5	1	1	1	0.5	0.5	0.5	1	0.25	0.5	2	2	1	1
*Aspergillus fumigatus*	0.5	1	0.5	1	1	1	0.5	1	1	2	0.25	5	1	2	1	1
*Penicillium funiculosum*	0.25	0.5	0.5	0.5	0.25	0.5	0.12	0.25	0.12	0.25	0.12	0.25	1	2	0.5	0.5
*Penicillium aurantiogriseum*	0.5	1	1	2	0.5	1	1	2	0.5	1	0.5	1	2	4	1	1

Note: MIC = minimal inhibitory concentration; MBC = minimal bactericidal concentration; MFC = minimal fungal concentration. Mix 1: 50% *R. officinalis*, 20% *C. nobile*, 15% *L. nobilis*, 15% *J. regia*. Mix 2: 40% *F. vulgare*, 30% *S. nigra*, 30% *H. perforatum*. Mix 3: 50% *M. pulegium*, 25% *O. europaea*, 25% *V. vinifera*. Mix 4: 60% *M. cervine*, 20% *C. arvensis*, 20% *R. idaeus*. Mix 5: 60% *O. vulgare*, 10% *C. nobile*, 15% *L. nobilis*, 15% *J. regia*. Mix 6: 70% *T. mastichina*, 15% *L. nobilis*, 15% *J. regia.*
